# Real-Time Ellipsometry at High and Low Temperatures

**DOI:** 10.1021/acsomega.2c07438

**Published:** 2023-01-17

**Authors:** Deshabrato Mukherjee, Peter Petrik

**Affiliations:** † Institute for Technical Physics and Materials Science, Centre for Energy Research, Budapest 1525, Hungary; ‡ Doctoral School of Materials Sciences and Technologies, Óbuda University, Budapest 1034, Hungary; § Department of Electrical and Electronic Engineering, Institute of Physics, Faculty of Science and Technology, University of Debrecen, Debrecen 4032, Hungary

## Abstract

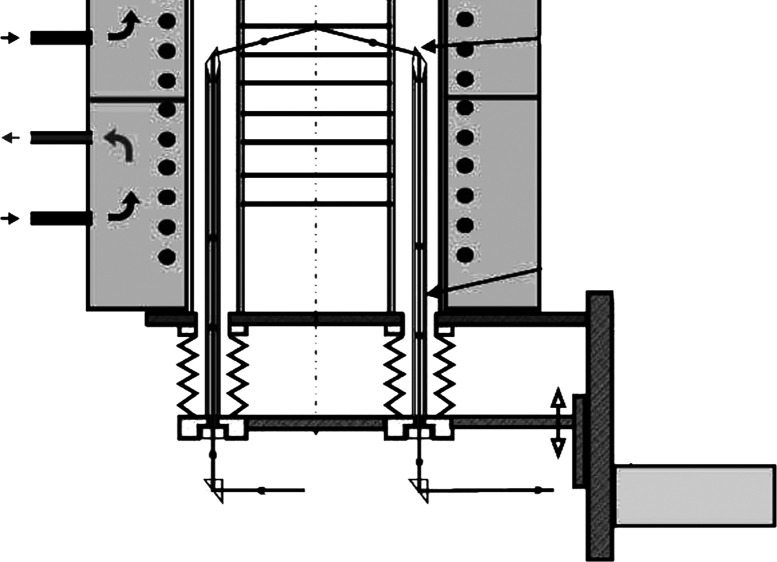

Among the many available real-time characterization methods, ellipsometry stands out with the combination of high sensitivity and high speed as well as nondestructive, spectroscopic, and complex modeling capabilities. The thicknesses of thin films such as the complex dielectric function can be determined simultaneously with precisions down to sub-nanometer and 10^–4^, respectively. Consequently, the first applications of high- and low-temperature real-time ellipsometry have been related to the monitoring of layer growth and the determination of optical properties of metals, semiconductors, and superconductors, dating back to the late 1960s. Ellipsometry has been ever since a steady alternative of nonpolarimetric spectroscopies in applications where quantitative information (e.g., thickness, crystallinity, porosity, band gap, absorption) is to be determined in complex layered structures. In this article the main applications and fields of research are reviewed.

## Introduction

**Figure 1:**
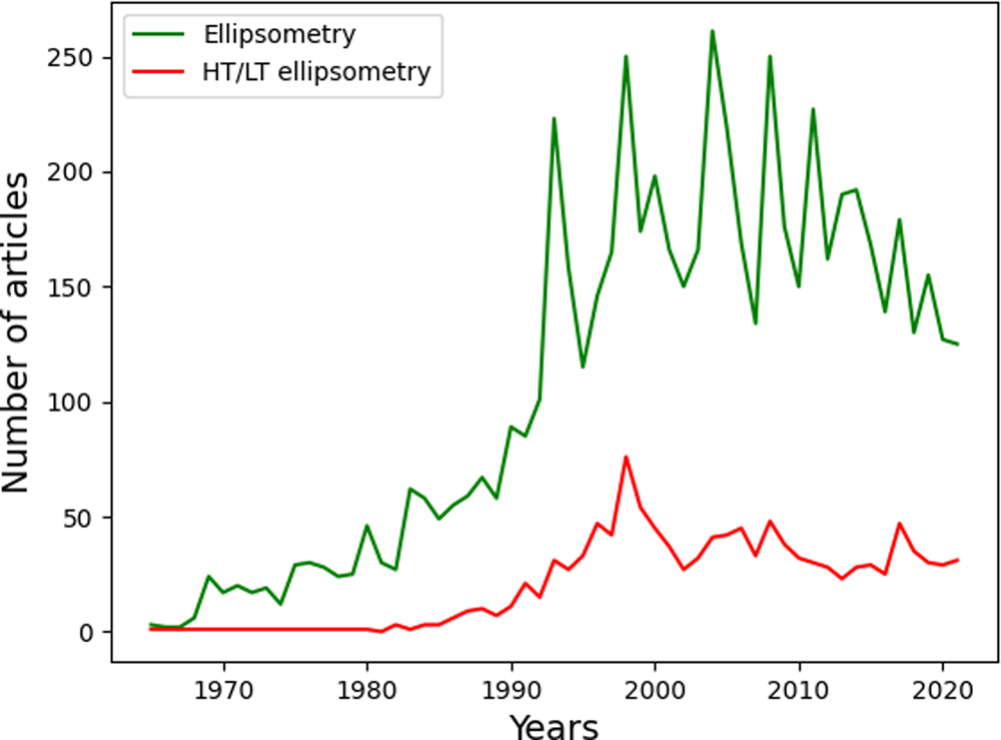
Number of articles containing the words “ellipsometry” or [“ellipsometry” and (“real time” or “in situ”) and “temperature”] in the title, abstract, or keywords, the latter denoted by “HT/LT ellipsometry” in the legend.

Ellipsometry measurements have already been made since the final decades of the 19th century pioneered by P. Drude.^1,2^ The measurement performed by B. Pogany in 1916 can already be considered as a multi-wavelength measurement.^3^ The term “ellipsometer” was coined in the article by A. Rothen in 1944 studying biomaterials on metal surfaces^4^ revealing sub-nanometer sensitivity. The reason for the early appearance of this technique is that, by using ellipsometry, high sensitivity can be achieved without a coherent light source and any other very expensive and sophisticated components. The most important hardware component has been the computer, which serves both as a control for the measuring device and as a tool for analyzing the data, since most ellipsometers are polarization modulation devices computing the measured values by analyzing the temporal line shapes of intensity signals. The need for computation is the result of the increase in the number of publications in the field of ellipsometry, which started to accelerate in the 1980s (Figure 1), coinciding with the era of affordable computation becoming available worldwide.

Today, the range of applications of ellipsometry has diversified to basic research in physical sciences, semiconductors, and data storage solutions as well as biosensor, communication, flat panel displays, and optical coating industries. Since the 1960s, ellipsometry has been implemented to provide the sensitivity necessary to measure nanometer-scale layers used in microelectronics resulting in increased interest at a steady rate in the field. In the 1980s, a rapid increase can be observed in both the development and the applications of ellipsometry (Figure 1). The real-time capabilities of ellipsometry were already utilized in the early 1980s.^5^

In this work, the presented studies of high-temperature (HT) or low-temperature (LT) ellipsometry are organized in three major groups: (i) instrumentation, (ii) monitoring of HT or LT processes, and (iii) determination of the reference dielectric functions at elevated or low temperatures (T). We exclude those investigations that focus on the ex situ characterization of the effect of HT (annealing) on the materials or structures and only deal with articles that measure real time at HT or LT. The number of ex situ characterizations is high, because both annealing and optical characterizations are basic methods in material processing and characterization. Numerous material properties that can be modified by annealing (e.g., band gap, crystallinity, porosity) can sensitively be measured and followed by optical methods.

We are only concerned here with real-time ellipsometry applications at temperatures higher or lower than room temperature (RT). Consequently, real-time optical measurements other than ellipsometry and real-time ellipsometry measurements at RT are excluded. Even so, looking at Figure 1, it is obvious that it is nearly impossible to include all the publications in the field in such a short review. Therefore, we only discuss a few significant achievements categorized by their type of applications and materials. In Table 1 we specify the topic and materials of all the papers discussed in this review ordered by the year of the work. We also include studies on processes at HT even if the temperatures have not been changed or the temperature dependence has not been investigated in real time (e.g., monitoring of growth at a given temperature).

**Table 1:** Summary of Publications on Real-Time Ellipsometry at HT and LT Ordered by Time^a^

Article	Topic	Material	T (K)
J.C. Miller^15^ 1969	Optical properties	Metals	1873
Y.J. van der Meulen^6^ 1974	Instrumentation	Si	RT-1450
E.A. Irene^39^ 1976	Oxidation	Si	1053–1253
D.E. Aspnes^142^ 1977	Optical properties	Ge	300, 1073
E.A. Irene^40^ 1977	Oxidation	Si	1053–1253
J.B. Theeten^5^ 1981	Monitoring of growth	Thin films	1533
E.A. Irene^41^ 1982	Oxidation	Si	873–1273
P. Lautenschlager^134^ 1985	Optical properties	Si, Ge	0–1000
H.Z. Massoud^42^ 1985	Monitoring of growth	SiO2	nan
S. Logothetidis^139^ 1986	Optical properties	GeS	84–500
A.M. Antoine^46^ 1987	Monitoring of growth	Amorphous Si and Ge	523
R.D. Frampton^44^ 1987	Oxidation	Silicides	973–1073
P. Lautenschlager^144^ 1987	Optical properties	Si	30–793
N.M. Ravindra^43^ 1987	Oxidation	Si	1073
J.C. Miller^15^ 1969	Optical properties	Metals	1873
Y.J. van der Meulen^6^ 1974	Instrumentation	Si	RT-1450
E.A. Irene^39^ 1976	Oxidation	Si	1053–1253
D.E. Aspnes^142^ 1977	Optical properties	Ge	300, 1073
E.A. Irene^40^ 1977	Oxidation	Si	1053–1253
J.B. Theeten^5^ 1981	Monitoring of growth	Thin films	1533
E.A. Irene^41^ 1982	Oxidation	Si	873–1273
P. Lautenschlager^134^ 1985	Optical properties	Si, Ge	0–1000
H.Z. Massoud^42^ 1985	Monitoring of growth	SiO2	nan
S. Logothetidis^139^ 1986	Optical properties	GeS	84–500
A.M. Antoine^46^ 1987	Monitoring of growth	Amorphous Si and Ge	523
R.D. Frampton^44^ 1987	Oxidation	Silicides	973–1073
P. Lautenschlager^144^ 1987	Optical properties	Si	30–793
N.M. Ravindra^43^ 1987	Oxidation	Si	1073
A. Bjørneklett^24^ 1988	Optical properties	Superconductor	80, 300
F. Lukeš^7^ 1988	Surface monitoring	GaAs	293–474
S. Andrieu^80^ 1989	Monitoring of growth	Sb	998
S. Kumar^47^ 1989	Surface monitoring	Amorphous Si	453
I. An^48^ 1990	Growth control	Si	573
D.E. Aspnes^8^ 1990	Growth control	Al_*x*_Ga_1.*x*_As	873
S. Matsuda^158^ 1990	Thickness measurement	Alloy600	293–368
I. An^49^ 1991	Optical properties	Si	573
T. Aoki^135^ 1991	Optical properties	Si	0–800
Y.Z. Hu^82^ 1991	Cleaning	Si	773
H. Yao^81^ 1991	Surface property	GaAs	850
D.E. Aspnes^86^ 1992	Monitoring of growth	AlGaAs	873
A.V. Boris^35^ 1992	Optical properties	Superconductor	10–200
R.W. Collins^97^ 1992	Monitoring of growth	Diamond	RT-1073
R.H. Hartley^87^ 1992	Monitoring of growth	CdHgTe	443–473
Y.Z. Hu^83^ 1992	Etching	Si	773
H.V. Nguyen^114^ 1993	Optical properties	Al	573
G. Vuye^136^ 1993	Optical properties	Si	293–723
T.T. Charalampopoulos^16^ 1994	Instrumentation	Thin films	RT-2573
R. Droopad^?^ 1994	Growth control	GaAs	873–1008
J. Humlicek^33^ 1994	Far IR optical properties	Superconductor	20–300
C.H. Kuo^140^ 1994	Optical properties	GaAs	RT-923
H.V. Nguyen^51^ 1994	Monitoring of growth	Si	532
R.K. Sampson^153^ 1994	Temperature measurement	Si	RT-1173
S. Yukioka^99^ 1994	Monitoring of growth	Polymer	443–483
J.T. Zettler^84^ 1995	Monitoring of growth	GaAs	773
Y.Z. Hu^52^ 1995	Monitoring of growth	Si	973
K. Kamarás^154^ 1995	Low temperature infrared	Perovskite	100–300
S. Trolier-McKinstry^98^ 1995	Annealing	Ferroelectric	RT-873
A. Cezairliyan^17^ 1996	Instrumentation	Metals	RT-2800
S.C. Deshmukh^91^ 1996	Monitoring of growth	SiO2	RT-538
Y.Z. Hu^56^ 1996	Monitoring of growth	Si	1123
A. Kussmaul^89^ 1997	MOCVD monitoring	AlGaAs, InGaAs	873–973
E. Steimetz^88^ 1997	Monitoring of growth	InAs	725–825
M.S. Thomas^155^ 1997	Optical properties	Vanadium oxides	RT-300
M. Zorn^141^ 1997	Optical properties	InP	RT-875
C. Basa^55^ 1998	Monitoring of growth	Si	873–933
R. Henn^26^ 1998	Synchrotron far-infrared	Superconductor	10–300
B. Johs^67^ 1998	Growth control	Hg_1–*x*_Cd_*x*_Te	293–523
J. Koh^62^ 1998	Monitoring of growth	Si	473
J. Lee^10^ 1998	Instrumentation	Si	1085
W. Lehnert^18^ 1998	Integration in vertical furnace	SiO_2_	1200
J. Šik^137^ 1998	Optical properties	Si	300–1200
M. Wakagi^50^ 1998	Phase transition	Si	853–898
V.A. Yakovlev^70^ 1998	Annealing	Si	1023–1373
S. Krishnan^19^ 1999	Phase transition	Metals	RT-2500
Y. Ohmasa^20^ 1999	Wetting phenomena	Mercury-sapphire	1623–1773
S. Yamamoto^32^ 1999	MOCVD monitoring	Superconductor	923
H. Fujiwara^61^ 2000	Monitoring of growth	Si	473
B. Gallas^90^ 2000	Oxidation	Si	373–673
A. von Keudell^53^ 2000	Monitoring of growth	Amorphous C	320
J.W. Klaus^115^ 2000	Monitoring of growth	WN	600–800
P. Petrik^58^ 2000	Integration in vertical furnace	Polysilicon	900
L. Pichon^131^ 2000	Transport properties	Zr	973–1073
J.A. Zapien^28^ 2000	Instrumentation	Thin films	523
P. Petrik^29^ 2001	Vertical furnace	Polysilicon	873
P. Petrik^59^ 2001	Crystallization	Si	873
R.I. Sheldon^21^ 2001	Optical properties	Ce	1700–2130
M. Tinani^71^ 2001	Phase transition	NiSi	623–1023
D. Apitz^100^ 2003	Electro-optic transition	Dye-doped organic	400
J. Backstrom^25^ 2004	Optical properties	Superconductor	20–325
A.V. Boris^34^ 2004	Spectral weight shift	Superconductor	30–300
M. Brown^27^ 2004	Instrumentation	Liquids	293–323
A. Deyneka^93^ 2004	High temperature effects	ZnLiO	793
Z.V. Feng^102^ 2004	Polyelectrolyte adsoption	Lipid bilayer	283-213
O. Bonaventurová Zrzavecká^101^ 2004	Optical properties	Polymer	300–473
S. Gupta^57^ 2005	Monitoring of growth	Si	323–788
G. He^129^ 2005	Oxidation	Zr	873–1173
X. Li^156^ 2005	Optical properties	PtOx	RT-973
A. Lyapin^123^ 2005	Oxidation	Zr	373–773
S.Y. Choi^111^ 2006	Phase transition	Titania	573–823
L.P.H. Jeurgens^121^ 2006	Oxidation	Zr	373–773
D.H. Levi^63^ 2006	Monitoring of growth	amorphous Si	363–713
A.V. Osipov^45^ 2006	Monitoring of growth	SiO_2_	308–473
O. Santos^103^ 2006	Monitoring of growth	Protein	313–367
M.S. Vinodh^122^ 2006	Oxidation	MgAl	304
B. Berini^157^ 2007	Optical properties	Conductive oxide	300–923
P.C. Wu^117^ 2007	Tuning	GaAs	RT-873
C. Eitzinger^30^ 2008	Monitoring	Dielectrics	nan
J.D. Bass^112^ 2008	Crystallization and sintering	Titania	923
K. Boukheddaden^68^ 2008	Phase transition	Charge transfer solids	150–400
J. Li^65^ 2008	Monitoring of growth	CdTe, CdS, CdTe_1–*x*_S_*x*_	418–593
N.J. Podraza^64^ 2008	Monitoring of growth	Si_1–*x*_Ge_*x*_	473–533
F. Reichel^124^ 2008	Oxidation	Al	350–640
F. Reichel^159^ 2008	Oxidation	Al	350–600
Z.M. Wang^69^ 2008	Phase transition	a-Si/Al	438–1023
A. Nebojsa^130^ 2008	Optical properties	Steel	300–923
G. Demirel^104^ 2009	DNA sensor	Polymer	298–318
E. Panda^127^ 2009	Oxidation	AlMg	300–485
A. Hadjadj^54^ 2010	Plasma interaction	Amorphous Si	373–523
E. Panda^128^ 2010	Oxidation	AlMg	300–610
G. Bakradze^132^ 2011	Oxidation	Zr	300–450
K. Boukheddaden^72^ 2011	Switching property	Molecular solid	296–383
A. Clough^106^ 2011	Phase transition	Polymer	300–400
C. Giannetti^36^ 2011	High-energy excitations	Superconductor	10–110
B. Berini^160^ 2012	Magnetic phase transition	Magnetic material	1000
K. Ide^74^ 2012	Relaxation	InGaZnO	RT-873
M. Koubaa^94^ 2012	Phase transition	Organic material	228–428
S.A. Little^120^ 2012	Phase transition	Ag	773
G.F. Malgas^105^ 2012	Phase separation	Polymer-fullerene	523
Y.K. Seo^73^ 2012	Phase transition	Phase change material	300–623
T. Jung Kim^143^ 2013	Optical properties	InSb	31–675
Y. Li^37^ 2013	Photon scattering	Superconductor	10–300
M. Schmid^22^ 2013	Optical properties	Au, Ag	1700
S. Tripura Sundari^148^ 2013	Optical properties	Ag	300–650
M. Rössle^95^ 2013	Optical properties	Perovskite	4–700
W. Ogieglo^107^ 2014	Glass transition	Swallen polymer	283–343
G. Rampelberg^75^ 2014	Phase transition	Vanadium oxides	RT-383
T. Karaki^96^ 2015	Optical properties	Piezoelectric	300–723
K. Weller^125^ 2015	Oxidation	Al_0.44_Zr_0.56_	773–833
D. Hrabovsky^79^ 2016	Surface monitoring	Strontium Titanate	300–1000
B.A. Humphreys^109^ 2016	Transition	Polymer brushes	293–318
K. Weller^126^ 2016	Oxidation	Al_*x*_Zr_1–*x*_	623–673
X. Yi^60^ 2016	Crystallization process	Ge_60_Te_40_	RT-623
J.A. Briggs^116^ 2017	Optical properties	TiN	RT-1531
B.K. Choi^23^ 2017	Band gap	MoSe_2_	1123
T.J. Murdoch^108^ 2017	Thermo-responsitivy	Polymer	283–323
H. Reddy^118^ 2017	Optical properties	Plasmonic	300–900
J. Sun^76^ 2017	Phase transition	Vanadium oxides	277–368
Y. Qian^92^ 2018	Oxidation	InSb/GaAs	293–573
B. Hajduk^110^ 2020	Phase transition	Polymer	303–500
Y.A. Aleshchenko^38^ 2021	Transport properties	Superconductor	5–300
Y. Liu^147^ 2021	Optical properties	AlN	RT-860
L. Pósa^77^ 2021	Phase transition	Vanadium oxides	340
M.A. Green^?^ 2021	Optical properties	Si	249–473
S. Bin Anooz^78^ 2022	Phase transition	NaNbO_3_	823
J. Budai^149^ 2022	Optical properties	Au, Ag	330–420

a Only the name of the first author is given, with the corresponding reference and year in the first column.

Finally, in the majority of the articles the phrases “in situ” and “real time” are used more or less as synonyms. “In situ” is used if the integration of the measurement into a process is emphasized, whereas “real time” is used if the simultaneous measurement during the process is in focus. We use “real time” for both cases because it also implies that the characterization technique is integrated into the processing device.

## Instrumentation

The majority of the real-time measurements presented in this review are based on homemade equipment, because commercial heat cells have not been available during most of the covered period of time. Many of the investigations utilize single-wavelength ellipsometry, which is sufficient in many cases to understand complex phenomena such as the oxidation of Si^6^ or the evolution of surface roughness.^7,8^ However, the development of spectroscopic ellipsometry (SE)^9^ and the rotating compensator version^10^ (later also double rotating compensator ellipsometry for the full Muller matrix analysis^11−13^) have substantially accelerated the development of the field. Rotating compensator ellipsometry is not only more suitable for real-time investigations but, due to the multichannel approach (measurement at each wavelength simultaneously at the same sensitivity—supported by the rotating compensator approach), the measurement time can also be decreased to the millisecond range while maintaining the spectroscopic capabilities.^14^

A few of the developed instruments give access to ultrahigh temperatures. For example, J.C. Miller^15^ measured the optical properties of seven metals up to *T* = 1873 K. T.T. Charalampopoulos et al.^16^ developed an HT ellipsometer to measure metal surfaces. A. Cezairliyan et al.^17^ utilized spectral radiometry and laser polarimetry to investigate Mo and W surfaces up to *T* = 2800 K. The device developed by J. Lee et al.^10^ is capable of monitoring the growth of thin films up to *T* = 1085 K. W. Lehnert et al.^18^ measured the oxidation of Si for *T* = 293 → 1200 K. S. Krishnan et al.^19^ applied high-speed laser polarimetry for the noncontact determination of phase transformation in metals and alloys up to *T* = 2500 K. Y. Ohmasa et al.^20^ investigated wetting phenomena at Hg-sapphire interfaces for *T* = 1623 → 1773 K. R.I. Sheldon et al.^21^ measured the optical properties of liquid Ce in the range of *T* = 1700 → 2130 K using electromagnetic levitation in order to avoid contamination during the process. M. Schmid et al.^22^ measured the optical properties of metals up to *T* = 1700 K. The band gap of MoSe_2_ was determined by Choi et al.^23^ at *T* = 1123 K.

Measurements conducted at ultralow temperatures also require special hardware and attention to the details. For the low-temperature measurements reported by Bjorneklett et al.^24^ the samples were held in a vacuum cell with a cryostat. During the low-temperature experiments the sample chamber was filled with oxygen at a pressure of 15–25 kPa in order to avoid the condensation of oxygen onto the surface of the sample. Furthermore, an oxygen background atmosphere was chosen to avoid oxygen depletion of the Y–Ba–Cu−O sample surface during measurement at 80 K. To avoid small freeze-outs on the sample surface, Bäckström et al.^25^ employed a measurement protocol with thermal cyclings between 10 K and room temperature between each pair of measured temperature points. R. Henn et al.^26^ evacuated the total volume of the Fourier spectrometer, the prechamber, and the ellipsometer chamber simultaneously in order to eliminate spurious absorption by air molecules. The sample chamber was separated by an additional lid, which allowed them to reach a pressure of about 10^–6^ mbar in the cryostat.

**Figure 2:**
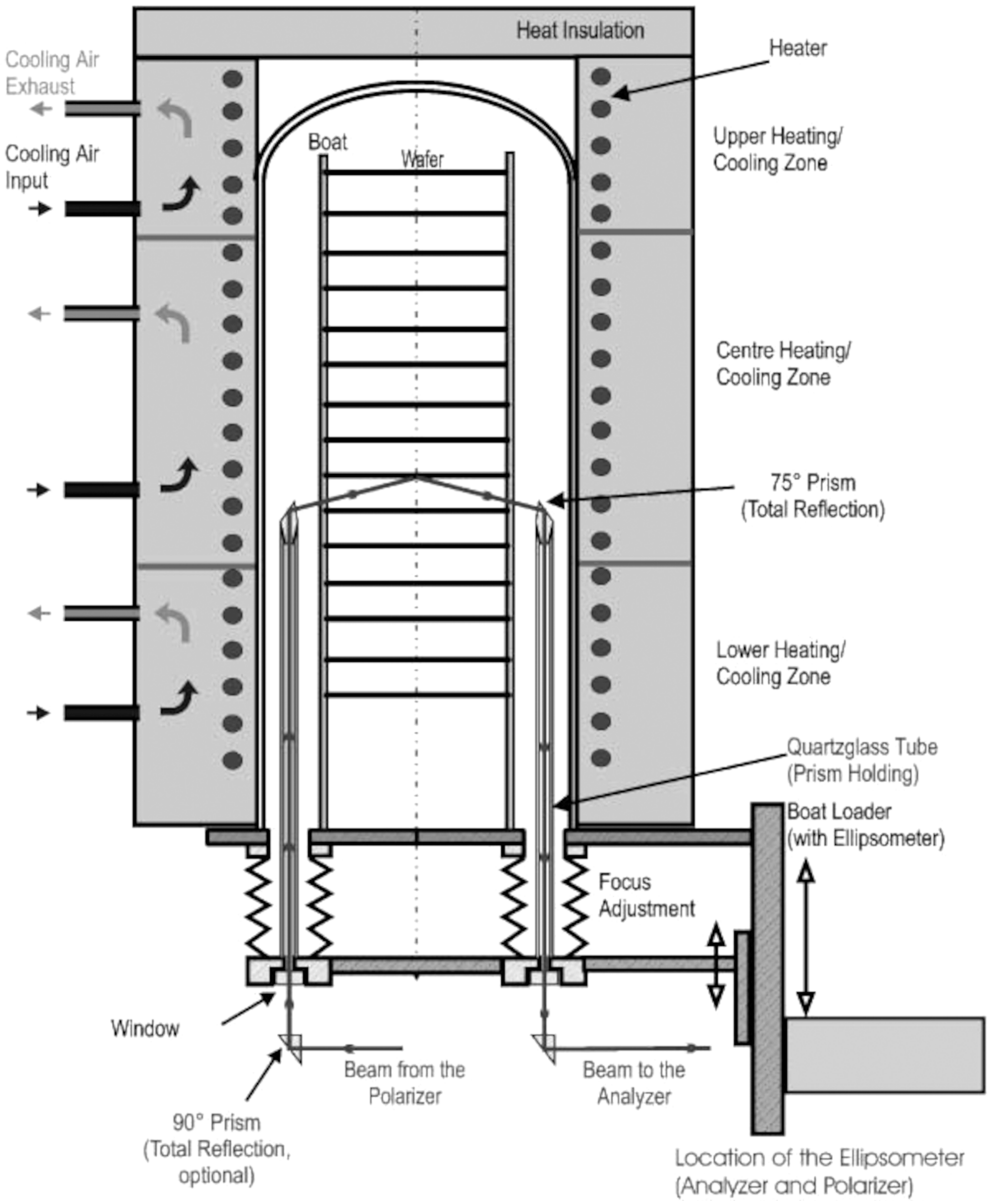
Integration of SE in a vertical furnace. Reprinted with permission from ref (29). Copyright 2001 Elsevier.

There have been special applications such as the combination of ellipsometry with other methods: M. Brown et al.^27^ built an ultrastable oven for the HT investigation of liquid surfaces using X-ray reflectometry and ellipsometry. Other examples include the high photon energy SE by J.A. Zapien et al.^28^ and the demonstration of SE in an industrial environment, integrating it into a vertical furnace by W. Lehnert et al.^18^ to follow layer growth during batch processing (Figure 2).^29^ Integration of SE in a chemical vapor deposition tool has been demonstrated by C. Eitzinger et al.^30^ J. Humlicek^31^ proposed a general scheme of analyzing the film growth in this tool using a series of in situ SE spectra in a closed-loop system.

## Investigation of Processes

### Semiconductors, Superconductors, and Related Materials

A large part of the LT SE studies is related to the characterization of superconducting materials. A. Bjorneklett et al.^24^ determined the optical properties of superconductor material Y–Ba–Cu−O. S. Yamamoto et al.^32^ monitored metal organic chemical vapor deposition (CVD) processes of superconductor materials at depositions up to *T* = 923 K. J. Humlicek et al.^33^ investigated superconducting materials at *T* = 20 → 300 K. R. Henn et al.^26^ used synchrotron radiation far-infrared ellipsometry to determine the out-of-plane response of the HT superconductor La_2–*x*_Sr_*x*_CuO_4_. The properties of the YBa_2_Cu_3_O_6.9_ HT superconductor (superconducting transition *T* = 92.7 K) were investigated by wide-band (0.01–5.6 eV) SE.^34^ The SE data provided real-time information on the optical self-energy in the normal and superconducting states. The optical conductivity σ, defined as ϵ(ω) = ϵ_1_(ω) + *iϵ*_2_(ω) = 1 + 4*πiσ*(ω)/ω (where ϵ and ω denote the dielectric function and the angular frequency, respectively), reveals a distinct feature at the superconduction transition temperature.^34^ Optical properties of cuprite superconductors have been measured by A.V. Boris et al.^35^ and J. Backstrom et al.^25^ C. Gianetti et al.^36^ revealed high-energy electronic excitations in superconducting cuprates. Li et al.^37^ investigated doping-dependent photon scattering resonance in the HT superconductor by Raman scattering and ellipsometry. Transport properties in HT superconductor BaFe_1.91_Ni_0.09_As_2_ have been studied by J.A. Aleshenko et al.^38^

Understanding the growth of oxide on Si has been one of the major issues of microelectronics from the dawn of the technology. The kinetics of oxide growth has been studied by E. Irene et al.^39,40^ already in the late 1970s using real-time SE, followed by several other studies of the same group,^41−43^ also for silicides.^44^ The real-time measurement of the oxidation of Si has also been demonstrated in a vertical furnace that has a smaller-sized system along with better contamination control.^18,29^ Laser-induced oxidation has been investigated by A.V. Osipov et al.^45^ for *T* = 308 → 473 K.

**Figure 3:**
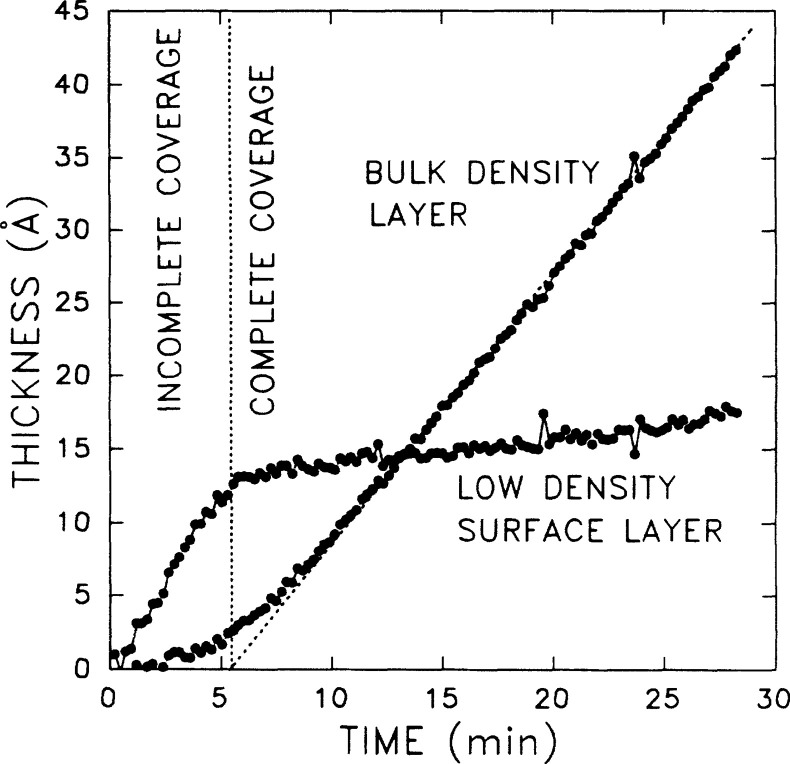
Evolution of amorphous Si surface roughness and bulk layer thickness during magnetron sputtering. Reprinted with permission from ref (48). Copyright 1990 The American Physical Society.

Real-time monitoring and control of thin-film growth for photovoltaic applications is one of the key topics of HT SE, in which the temperature of the substrate is a critical process parameter. The majority of the studies deal with amorphous or microcrystalline Si and Ge, such as the growth of glow-discharge deposited amorphous Si (a-Si) and Ge (a-Ge) comparing the growth at RT and *T* = 523 K, developing models for the formation of nanoroughness,^46^ or the formation of amorphous Si on transparent conductive oxides at *T* = 453 K.^47^ The growth of amorphous Si was followed by real-time SE at *T* = 573 K,^48,49^ and the crystallization of amorphous Si was observed at *T* = 853 → 898 K.^50^ SE was proven to be a unique tool to reveal and optimize nucleation and a roughness layer separate from the bulk layer during thin-film growth (Figure 3, ref (48)), which greatly contributes to the identification and optimization of microcrystalline phases for photovoltaic applications.^51^ Hu et al.^52^ measured the incubation time for Si nucleation on SiO_2_ in a rapid thermal process at *T* = 973 K. The interaction between methyl radicals and atomic H during the growth of amorphous hydrogenated carbon films has been studied by A. von Keudell et al.^53^ for *T* = 320 K, whereas the interaction with H plasma has been investigated in detail by A. Hadjadj et al.^54^ at *T* = 373 → 523 K.

Rapid thermal chemical vapor deposition was used by C. Basa et al.^55^ to create polycrystalline Si layers at *T* = 1123 K^56^ and *T* = 873 → 933 K. Hot wire deposition has also been studied by Gupta et al.^57^ at *T* = 323 → 788 K. W. Lehnert et al.^18^ and P. Petrik et al.^58^ demonstrated the integration of real-time SE in a vertical furnace by the example of thermal oxidation of Si and crystallization of a-Si, respectively.^29,59^ The crystallization of Ge_60_Te_40_ has also been investigated for *T* = 293 → 623 K.^60^ The growth of amorphous Si films has been monitored by H. Fujiwara et al.^61^ using real-time SE at *T* = 473 K. The same group has demonstrated the applicability of real-time ellipsometry for the development of thin films for solar applications in numerous publications, see, e.g., ref (62). D.H. Levi et al.^63^ also developed real-time SE for the optimization of Si-based photovoltaic structures during hot wire chemical vapor deposition at *T* = 363 → 713 K. Silicon-based compound semiconductor structures have also been studied, such as the growth of graded Si_1–*x*_Ge_*x*_ films followed using real-time ellipsometry by N. Podraza et al.^64^ at *T* = 473 → 533 K for the substrate. The same group, focusing on the investigations of photovoltaic materials, published in the same year a study on the deposition and growth of CdTe, CdS, and CdTe_1–*x*_S_*x*_ by J. Li et al.^65^ using real-time SE at *T* = 418 → 593 K. A parametric B-Spline model^66^ has been developed by B. Johs et al.^67^ for Hg_1–*x*_Cd_*x*_Te to control the composition during molecular beam epitaxial growth.

The capability of SE to determine not only thicknesses but also both the real and imaginary parts of the dielectric function simultaneously has been utilized in many phase-transition studies in charge transfer solids (*T* = 150 → 400 K),^68^ crystallization of amorphous Si^29,59^ also in the presence of Al,^69^ annealing of Si,^70^ NiSi (*T* = 623 → 1023 K),^71^ the switchable molecular solid RbMn[FeCN_6_] (*T* = 150 → 400 K),^72^ Ge_2_Sb_2_Te_5_ phase changing material (*T* = 293 → 623 K),^73^ relaxation in a-InGaZnO,^74^ and phase change in vanadium oxides.^75−77^ S. Bin Anooz et al.^78^ determined the phase transition in epitaxial NaNbO_3_ films grown under tensile lattice strain on the (110) DyScO_3_ substrate up to *T* = 823 K. The *n* is measured at an energy of 3.2 eV, i.e., near the band gap of 3.9 eV, to best observe variations with phase transitions and structural changes. At RT, monoclinic a1a2 ferroelectric phase with exclusive in-plane electrical polarization and at *T* = 523 → 573 K depicts a ferroelectric-to-ferroelectric phase transition. At around *T* = 773 K, a further transition to the paraelectric phase was observed.

Formation and features of surface structures have been studied on GaAs (*T* = 474 K)^7^ and strontium titanate surfaces (*T* = 293 → 1000 K).^79^ S. Andrieu et al.^80^ followed Sb adsorption on Si(111) at *T* = 998 K revealing adsorption/desorption kinetics. H. Yao and P.G. Snyder^81^ have presented real-time SE data from both oxidized and unoxidized surfaces of GaAs(100) at elevated temperature in ultrahigh vacuum. Real-time data showed the desorption of native oxide at approximately *T* = 850 K causing a surface roughening and degradation. Cleaning of the surface of Si wafers has been studied by Hu et al.^82^ showing that the residual damage can be monitored by SE. This group also studied the etching of Si surface by Ar and H ions revealing a saturation of the damage layer with the etching time in case of Ar.^83^

**Figure 4:**
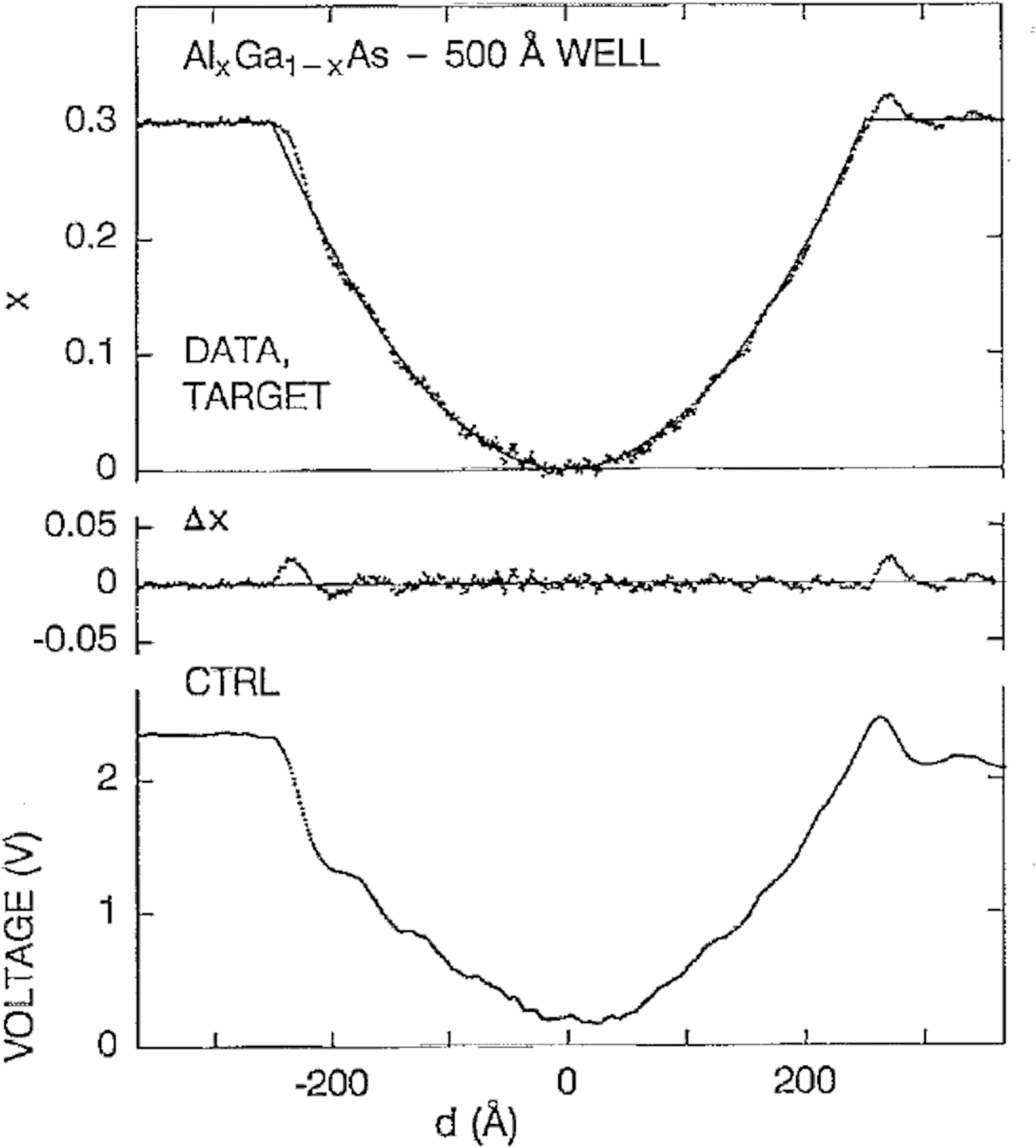
Composition control during growth of an Al_*x*_Ga_1–*x*_As layer to create a parabolic quantum well. Reprinted with permission from ref (86). Copyright 1992 AIP Publishing.

Thin film growth has been controlled for epitaxy of GaAs,^84^ Al_*x*_Ga_1–*x*_As^8,85^ (also with control for parabolic composition profile^86^), CdHgTe and CdTe/HgTe superlattices,^87^ InAs,^88^ and for metal organic CVD of AlGaAs and InGaAs (*T* = 873 → 973 K).^89^ The capabilities of SE for a precise composition control during deposition has been demonstrated by D.E. Aspnes et al.^86^ (Figure 4). B. Gallas et al.^90^ investigated the formation of oxide layer on Si for reflective dielectric mirror applications, whereas S.C. Deshmukh et al.^91^ monitored metal–organic vapor-phase epitaxy of GaN for optoelectronics. A versatility of other effects has also been investigated including oxidation of InSb/GaAs (*T* = 523 → 573 K)^92^ and Si (*T* = 1200 K)^18^ surfaces or HT effects in Li-doped ZnO.^93^

Perovskites, langasite, and other special crystal structures have been studied for a broad range of applications. M. Kouaba et al.^94^ explored the thermal properties of the perovskite slab alkylammonium lead iodide using real-time ellipsometry and numerous complementary methods. The thermal behavior of the excitonic absorption obtained by SE and PL showed a good quantitative agreement, but it was not possible to measure both the heating and cooling modes by SE due to the long data acquisition time (∼180 s) causing photodegradation of the material at HT. The ferroelectric ordering has been studied in SrTiO_3_ and BaTiO_3_ by Rössle et al.^95^ at *T* = 4 → 700 K, with a special emphasis on its influence on the direct band gap close to the ferroelectric transition. It has been shown that the anomalous T-dependent shift of the direct band gap of SrTiO_3_ is strongly affected by the Fröhlich electron–phonon interaction with the so-called soft mode that is at the heart of its quantum-paraelectric properties.

Piezoelectric materials of the langasite family have been investigated by T. Karaki et al.^96^ Nucleation of diamond has been monitored during filament-assisted CVD at substrate temperatures of *T* = 300 → 1073 K.^97^ S. Troiler-McKinstry et al.^98^ studied the annealing of sol–gel ferroelectric thin films to follow the crystallization process at *T* = 773 → 873 K.

### Dielectrics and Organic Materials

A large portion of real-time temperature-dependent ellipsometry studies on dielectrics includes polymers and organic materials. Compatibilization of immiscible polymer blends have been investigated by S. Yukioka et al.^99^ at *T* = 443 → 483 K. Orientational dynamics in dye-doped organic electro-optic materials has been investigated by Apitz et al.^100^ together with the temperature dependence of the phenomenon. It has been shown that the switching properties of the chromophores in a guest–host polymer composite based on Disperse Red 1 and poly(methyl methacrylate) hardly depends on the temperature. Bonaventurová-Zrzavecká et al.^101^ determined the temperature-dependent optical properties of an organic-inorganic polymer material poly(methyl-phenylsilane). They identified the onset of thermal degradation at *T* = 373 K. Below this temperature the optical response was reversible with an average shift of the lowest excitonic band of −8.5 × 10^–4^ eV/K. Lipid bilayer modification by polyelectrolyte adsorption was investigated by Z.V. Feng et al.^102^ using real-time ellipsometry. In this study, the melting temperature is lowered from 297 to 294 K of a phospholipid bilayer made from 1,2-dimyristoyl-*sn*-glycero-3-phosphocholine (DMPC) when added with a weak polyelectrolyte, poly(methacrylic acid) (PMA). A slight asymmetry is also observed upon PMA addition in the gel phase, further verified by other characterization procedures.

In a special tool and application O. Santos et al.^103^ monitored protein adsorption onto steel surfaces at *T* = 313 → 367 K, in which both the surface properties and the bulk solution conditions affected the adsorption rate. G. Demirel et al.^104^ used polymer layers on a Si wafer for DNA sensing. Here, a validation test was conducted at *T* = 298 and *T* = 318 K, below and above the lower critical solution temperature value, respectively, on the Si(001) platform that interacted with the complementary of the probe “immobilized” oligo or the noncomplementary model oligo. It was confirmed that the hybridization between the probe and the target within the medium can be modulated. G. F. Malgas et al.^105^ studied the temperature dependence of the phase separation in polymer–fullerene films. The study determined the optimum temperature to obtain the desired phase separation for solar cell application in P3HT:PCBM film, a methanofullerene derivative. The measurements using SE were made at multiple angles of incidence that showed a reduction in the electronic peaks of PCBM, causing an improved extinction coefficient and refractive index during annealing at 413 K.

**Figure 5:**
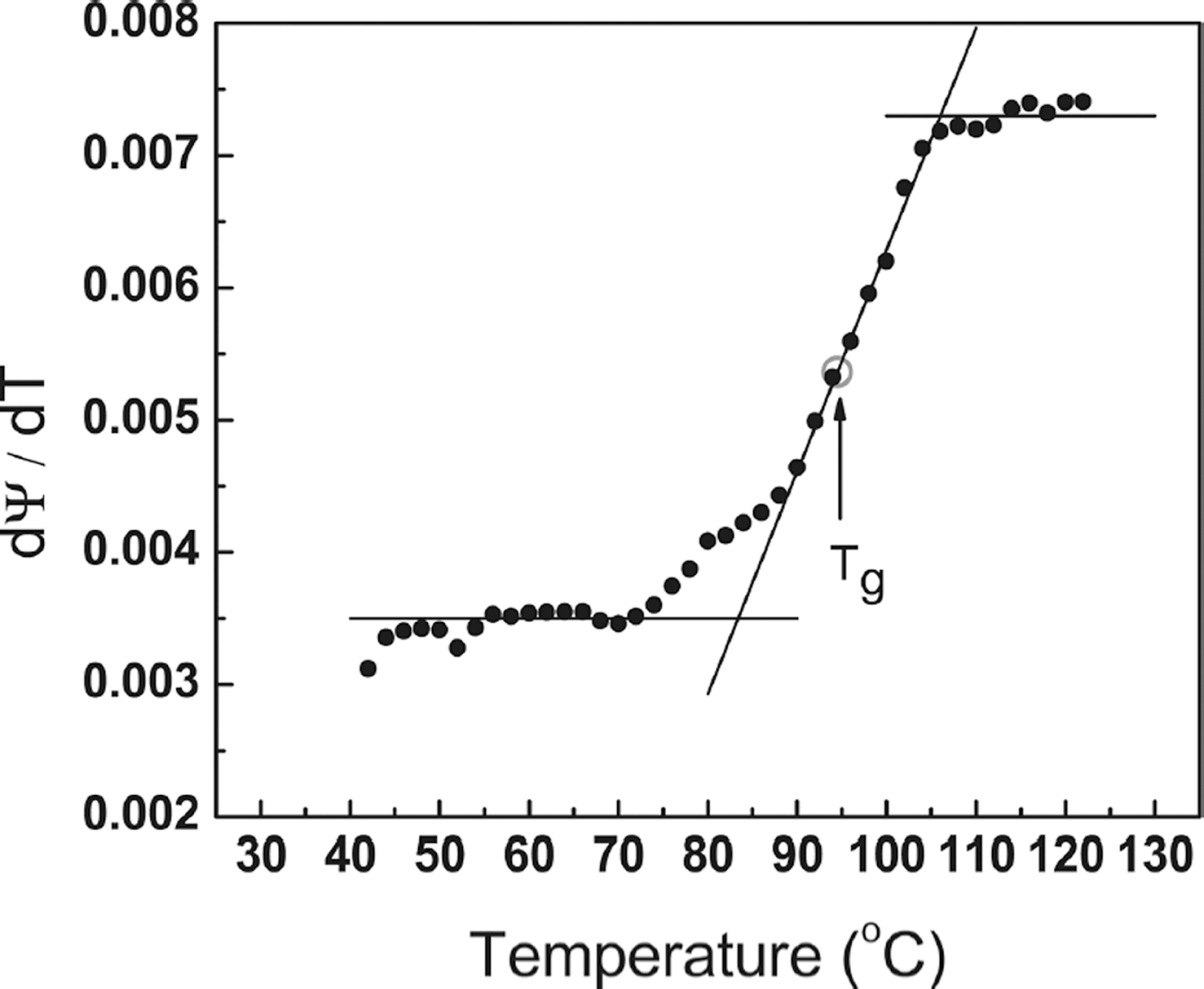
Derivative of the ellipsometric Ψ parameter as a funtion of *T* for the identification of the glass transition temperature (*T*_g_). Reprinted with permission from ref (106). Copyright 2011 The American Chemical Society.

Glass transition and thickness change has been investigated in polymers by A. Clough et al.^106^ for *T* = 300 → 400 K. Both the change of the optical properties and the thickness have been monitored by real-time ellipsometry determining the major features of the kinetics (Figure 5). Ogieglo et al.^107^ investigated the glass transition in swollen polymers (polystyrene). For SE studies, a temperature stabilization system that operates in the range of *T* = 283 → 333 K was equipped to the test cell. Thermal equilibrium was maintained within the system, as polymer chains and penetrant mobility are large above glass transition temperatures, whereas the solvent concentration in the swollen matrix reduces when the temperature is lowered. T.J. Murdoch et al.^108^ investigated enhanced ion effects in thermoresponsive polymer brushes by real-time ellipsometry. The thermoresponse of homo- and copolymer PMEO2MA brushes (size 540 ± 30 Å) in aqueous solution were characterized via different techniques. Ellipsometry measurements showed that the main impact of the addition of salt is a displacement of the overall temperature response along the temperature axis, where increase in thiocyanate concentration up to 250 mM shifted the response to higher temperatures, while increasing acetate concentration shifted the response to lower temperatures. B.A. Humphreys et al.^109^ investigated the thermoresponse of polymer brushes by the combination of SE and quartz crystal microbalance (QCM) for *T* = 293 → 318 K. HT ellipsometry has been reviewed recently for polymers by B. Hajduk et al.^110^

The formation of mesostructured nanocrystalline titania thin films has been monitored by both real-time SE and X-ray diffraction (XRD) revealing a perfect complementary character, with SE showing the thickness and the porosity and XRD determining the crystallinity.^111^ Bass et al.^112^ investigated the pyrolysis, crystallization, and sintering of titania films assessed by real-time thermal ellipsometry. It is used to determine the evolution of porosity and characterization of the influence of parameters such as heating schedule, initial film thickness, nature of the substrate, solution aging, presence of water during calcination, nature of the templating agent, and influence of additives in the calcination environment as a function of temperature. Romanenko et al.^113^ used real-time ellipsometry to determine characteristics of zirconia films formed on the surface of Zr during oxidation at *T* = 300 → 700 K.

### Metals, Conductive, and Related Materials

There have been a few studies by SE in the 1990s on the evolution of optical properties of metals in both solid and liquid forms. Al has been studied by Nguyen et al.^114^ up to *T* = 573 K. Phase transformation in metals has been measured by Krishnan et al.^19^ using HT real-time laser polarimetry^17^ at temperatures up to *T* = 2500 K. Wetting properties of the Hg-sapphire interface have been characterized using real-time ellipsometry at pressures and temperatures up to 144 MPa and *T* = 1773 K, respectively.^20^ It was found that the highly precise detection of the wetting layer was possible on comparison of the *R*_p_ and *R*_s_ reflections, along with confirmation of the prewetting transition via a 45° reflection measurement setup using a wedge-shaped sapphire rod. Metallic materials can also be used as diffusion barriers, e.g., against Cu. The atomic layer deposition (ALD) growth of WN, one kind of those materials, has been monitored by J.W. Klaus et al.^115^ to reveal a linear growth rate at *T* = 600 → 800 K. Another promising application of refractory metal nitrides is nanophotonics and plasmonics, for which the high-temperature optical properties are essential data. These have been determined for TiN by J.A. Briggs et al.^116^ for *T* = RT → 1531 K.

The temperature dependence of plasmonic materials is also a hot topic. Wu et al.^117^ have shown for *T* = RT → 873 K that the plasmonic properties of Ga nanoparticles can be tuned. Thermal stability has been revealed for Ag by H. Reddy et al.,^118^ which is a key factor in many other fields including solar materials.^119^ The melting temperatures and HT phase transitions in Ag have been measured by S.A. Little et al.^120^ up to *T* = 773 K. M. Schmid et al.^22^ measured the optical properties of Au and Ag at *T* = 1700 K parametrized by Lorentz oscillators. In the case of Au samples, the refractive index (*n*) increases with increasing temperature in the solid as well as in the liquid phase, and the absorption coefficient (*k*) depicts the influence of the cracking up of the debris layers at high temperatures above the melting point on the surface of the liquid metal sample, while upon heating the Au sample below the melting point, the surface of the sample changed from a smooth surface to a satin-like texture and back to smooth again. For a Ag sample, the experimental value of the *n* decreases with increasing temperature below the melting point.

**Figure 6:**
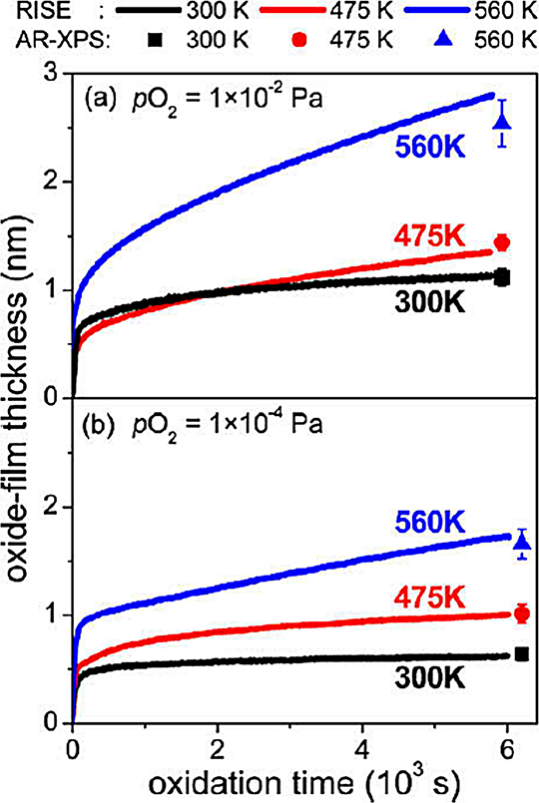
Film growth by real-time SE (lines) and XPS (symbols) during the oxidation of AlMg alloy. Reprinted with permission from ref (128). Copyright 2010 Elsevier.

The oxidation of metal surfaces has been measured by numerous techniques that involve ellipsometry. The group of E.J. Mittemeijer measured in real time the initial stages of oxidation of a range of crystalline metal surfaces. A few studies used real-time ellipsometry alone, such as investigating the growth of ultrathin oxides on Zr^121^ or MgAl alloys.^122^ Another way is the combination of different methods either separately, such as the combination of depth profiling Auger electron spectroscopy and SE for the study of Zr oxidation,^123^ passivation of Al surfaces,^124^ and AlZr alloys,^125,126^ or a simultaneous measurement such as the characterization of the surface by real-time SE and X-ray photoelectron spectroscopy (XPS) to investigate the initial stages of the oxidation of Zr,^123^ Al,^124^ and AlMg^127,128^ (Figure 6). G. He et al.^129^ oxidized Zr in thin-film form, where it was also studied by real-time SE at temperatures of *T* = 873 → 1173 K. The critical role of the sample surface in the HT optical properties of pure Fe and steel has been shown by A. Nebojsa et al.^130^ for *T* = RT → 923 K. The authors identified the influence of the increased temperature on the magnetic contribution to the electronic interband transitions. Pichon et al.^131^ revealed a complex mechanism during plasma nitridation of Zr at *T* = 973 → 1173 K. Oxidation on bare Zr substrates was performed at *T* = 375 → 773 K. At lower temperatures (423 K), oxidation stops after the first stage at a limiting thickness that increases with temperature (0.6 nm at 373 K; 0.7 nm at 423 K), while at *T* > 423 K a second stage of much slower, but continued, oxide-film growth occurs.^123^ The orientation-dependent oxidation kinetics has also been investigated on Zr by Bakradze et al.^132^ Romanenko et al.^113^ used real-time ellipsometry to create diffusion models for the oxidation of Zr.

## Determination of Reference Dielectric Functions

One of the most important materials of electronics is Si, the optical properties of which have been investigated at high temperature in both crystalline and amorphous forms. The interband structure in crystalline Si shows three sharp peaks that are blended into a single broad peak in the amorphous samples.^133^ P. Lautenschlager et al.^134^ (*T* = 0 → 1000 K), T. Aoki et al.^135^ (*T* = 0 → 800 K), G. Vuye et al.^136^ (*T* = 293 → 723 K), and J. Sik et al.^137^ (*T* = 300 → 1200 K) determined the dispersion of refractive index of Si, whereas the optical properties of amorphous Si have been determined by I. An et al.^49^ during deposition on HT substrates up to *T* = 573 K. A recent review with tabulated optical function of Si for photovoltaic applications has been published by M. A. Green^138^ for *T* = 249 → 473 K, which heavily relies on ellipsometric results.

The optical properties and the related electron band structure have been analyzed for a couple of semiconductors by several authors. The group of M. Cardona investigated numerous semiconductors at HT from the middle of the 1980s. The temperature dependence of the band gap of Si and Ge was investigated by Lautenschlager et al.^134^ for *T* = 0 → 1000 K. This work was followed by numerous studies by the same group on the fundamental band structure models of semiconductors and their dependence on the temperature, such as the investigations by Logothetidis et al. on GeS in the range of *T* = 0 → 1000 K.^139^ C.H. Kuo et al.^140^ measured the optical constants of GaAs from RT to *T* = 923 K, whereas M. Zorn et al.^141^ measured those of InP for *T* = RT → 875 K. B.K. Choi et al.^23^ measured the band gap of epitaxial MoSe_2_ at HT. D.E. Aspnes et al.^142^ determined the optical properties of Ge at *T* = 295 → 1073 K by utilization of a modified photometric polarimeter and ellipsometer system. At *T* = 1073 K, the sample with a dull orange glow was detected via the photomultiplier and started to strongly degenerate due to shrinking of the band gap and thermal excitation, where all structures are broadened and shifted to lower energy by as much as 0.4 eV. Temperature-dependent dielectric functions of InSb have been measured by T.J. Kim et al.^143^ in the photon energy range of 0.7–6.5 eV and *T* = 31 → 675 K. The critical point features have also been analyzed utilizing the second-derivative method.^144−146^ The optical properties of AlN films have been investigated at *T* = RT → 860 K by Y. Liu et al.^147^

References for metals have been determined in both liquid and solid forms. The optical properties of seven liquid metals have been measured by J.C. Miller^15^ in an early pioneer work in 1969 up to *T* = 1873 K. Optical properties of Ag have been measured by S. Tripura Sundari et al.^148^ in the photon energy range of 1.4–5.0 eV at *T* = 300 → 650 K, together with the thermo-optic coefficient using real-time SE. Temperature-dependent optical properties of Au have been determined to demonstrate experimentally that, upon optical excitation of the surface plasmon polaritons, a nonthermal electron population appears in the topmost part of the illuminated Au layer.^149^

It has also been demonstrated that ellipsometry and polarimetry are capable of measuring the optical properties of materials in the liquid state. It has not only been discussed for the case of liquid metals shown above^150^ but also for water. The temperature dependence of the optical properties of water has been determined by G. Abbate et al.^151^ in 1978 revealing an exponential behavior, replacing a previously developed transmission-based method by ellipsometry.^150^

As a special application, SE was used as a nonintrusive means of temperature measurement of Si wafer by Kroesen et al.^152^ for *T* = 300 → 373 K. The detailed database on the temperature dependence of Si can also be used as a tool for the determination of the temperature.^136^ This capability has been demonstrated by R.K. Sampson et al.^153^ using Si in the temperature range from RT to 1173 K. The benefit of using shorter wavelengths than the usual 632.8 nm was pointed out, increasing the resolution of the temperature determination.

Reference optical data have been determined and analyzed for many oxide materials. K. Kamaras et al.^154^ investigated the LT optical functions of SrTiO at *T* = 20 → 300 K. The optical properties of vanadium oxide have been measured by M.S. Thomas et al.^155^ including the phase-transition temperatures. Li et al.^156^ determined the optical properties of PtO_*x*_ at *T* = RT → 973 K. PtO_*x*_ is oxidized on heat treatment and then decomposes into Pt at 822 K. Condensation of porous Pt film occurs at *T* = 973 K for the samples with *x* > 1.3, where the surface roughness increases at *T* > 822 K. PtO_*x*_ changes to metallic Pt via oxidization, decomposition, and condensation at elevated temperatures. B. Berini et al.^157^ measured the reference dielectric function for the conductive oxide LaNiO_3_ at *T* → 923 K. A change in the optical constants as a result of change in temperature was observed for *T* = 513 → 673 K.

## Conclusions

Ellipsometry has been a widely used tool in the real-time characterization and monitoring of HT and LT processes since the 1960s. In the last decades, besides the initial application of superconducting, microelectronic, and semiconducting materials, new fields emerged including plasmonic, organic, and polymer applications. Similar to liquid metals in the early applications, now solid–liquid interfaces have also been investigated with temperature control and study of temperature effects. In many cases, vacuum chambers are replaced by small heat and liquid cells that can be used with table-top ellipsometers. Due to the sensitivity of SE to the crystalline order, the electron structure, and thickness of ultrathin films, an increase in applications is to be expected for 2D, perovskite, plasmonic, bio, and a range of other new materials.
